# Comparative Finite Element Evaluation of Polymeric and Metallic Bioresorbable Sinus Stents Under Quasi-Static Radial Compression

**DOI:** 10.3390/jfb17020083

**Published:** 2026-02-08

**Authors:** Wenyu Fu, Aiping Yang, Aike Qiao

**Affiliations:** 1 College of Robotics, Beijing Union University, Beijing 100027, China; jdtwenyu@buu.edu.cn; 2 Beijing Engineering Research Center of Smart Mechanical Innovation Design Service, Beijing 100027, China; 3 College of Chemistry and Life Science, Beijing University of Technology, Beijing 100124, China; 4 Beijing International Science and Technology Cooperation Base for Intelligent Physiological Measurement and Clinical Transformation, Beijing 100124, China

**Keywords:** biodegradable sinus stent, radial pressure, loading–unloading hysteresis, plastic deformation, finite element analysis

## Abstract

To address the issues of displacement and insufficient positional stability observed in the clinical use of the PROPEL Mini stent, this study investigates the influence of different biodegradable materials on the mechanical properties of the stent under the constraint of a fixed monofilament braided closed-loop geometry. Finite element analyses are conducted using Abaqus/Explicit to quantitatively evaluate the nonlinear mapping between nominal diameter, axial length, and radial pressure throughout a loading–unloading cycle. The results reveal that while axial behavior is consistent during compression, material-specific plasticity causes irreversible geometric sets in Mg alloy and PLGA models, whereas the PCL stent achieves total elastic recovery to its initial dimensions. During unloading, the Mg alloy stent recovers to a nominal diameter of 28 mm with a reduced axial length of approximately 22 mm, whereas the PLGA stent exhibits a much smaller recovery diameter of 14 mm with an axial length of approximately 23 mm. These post-release configurations directly determine the functional expansion range of the biodegradable stents after implantation. During unloading, the Mg alloy stent provides the highest radial pressure (peak 6.8 kPa) with a functional recovery range up to 26.5 mm, ensuring superior scaffolding stability. In contrast, while PCL achieves the widest recovery (52 mm), its radial pressure is clinically negligible (the maximum value is still less than 165 Pa), and the PLGA model exhibits both insufficient support and a restricted functional recovery limit (13 mm). By using high-strength materials such as Mg alloys, the radial anchoring force of the stent can be effectively enhanced without changing the existing structure, providing a scientific basis for solving clinical displacement problems.

## 1. Introduction

Functional endoscopic sinus surgery (FESS) is the core approach for treating chronic sinusitis, and the introduction of self-expanding PROPEL Mini stents has provided strong support for maintaining sinus ostium closure and reducing mucosal inflammation after surgery [1,2]. However, with the increasing clinical application of PROPEL Mini stents, their “Achilles’ heel”—the problem of unstable displacement and accidental expulsion after implantation—has gradually become prominent, becoming a key bottleneck limiting their long-term efficacy [3,4,5,6,7].

From a biomechanical perspective, the stability of a stent essentially depends on the mechanical balance between its radial force and the frictional force of the sinus mucosa. To improve stability, researchers have begun to explore alternative materials. For example, Lu et al. attempted to compare the contact characteristics of PLGA, PCL and Magnesium alloy during stent deployment and suggested that PCL might be a potential choice to avoid displacement [8]. Mechanical properties, particularly radial supporting capacity and material degradation, have been identified as pivotal factors in the success of bioabsorbable stents across various clinical applications. For instance, Magnesium-based biliary stents have been explored for their excellent biocompatibility and anti-tumor potential [9]. Similarly, computational frameworks have been developed to evaluate the radial force of 3D-printable airway stents [10] and the viscoelastic expansion behavior of PLLA coronary scaffolds [11], emphasizing the need for precise mechanical prediction during the early stages of implantation. However, most of these studies remain at a qualitative level and lack a quantitative characterization of the relationship between radial pressure and nominal diameter.

The lack of quantitative prediction stems from the complexity of mechanical modeling of braided structures. A classic analytical expression was proposed by Jedwab et al. in 1993, which quantifies how the radial pressure—defined here as the normal contact pressure exerted by the stent on the surrounding surface—exerted by a braided self-expanding stent varies as a function of its nominal diameter [12]. Unfortunately, this formula is based on the assumption of “independent helical spring coils” and is only applicable to open-coiled structures; in contrast, the PROPEL Mini stent uses a complex closed-cell monofilament braided structure [1,13], and the mutual constraints between its meshes lead to a highly geometrically nonlinear mechanical behavior.

However, evaluating the mechanics of complex braided structures like PROPEL Mini is challenging without a comprehensive understanding of the materials and optimized simulation strategies. Accurate pre-degradation mechanical testing, including stress–strain and relaxation data, is fundamental for predicting the load-carrying capacity of PLLA-based devices [14]. Furthermore, finite element analysis (FEA) has been proven as an efficient tool for evaluating the design and scaffolding time of biodegradable platforms, such as various Magnesium alloy stent geometries [15]. Despite these advancements in vascular and respiratory fields, a quantitative study specifically focusing on the compression and rebound mechanics of the PROPEL Mini’s closed-cell monofilament structure using multiple degradable material models remains absent in the current literature. We searched the mainstream databases such as PubMed and found that no studies can accurately describe the quantitative law of radial force variation with diameter during the compression and rebound process of the PROPEL Mini stent. Since the geometry of the PROPEL Mini stent is fixed after manufacturing, the only variable that seems to change its mechanical response is the material properties. However, it is unclear whether changes in the material properties will have a ripple effect. For example, will increasing radial pressure exacerbate axial elongation of the stent? Such axial deformation often corresponds to shear damage to the mucosa in clinical practice [16].

To address the aforementioned gaps, this paper employs explicit finite element analysis (Abaqus/Explicit) to systematically investigate the mechanical response of PROPEL stents made from PLGA, PCL, and Magnesium alloys during compression and rebound. We not only focus on the quantitative relationship between radial pressure and nominal diameter but also delve into whether the geometric characteristic of axial elongation possesses “material independence.” This study aims to provide theoretical criteria for the material selection and structural optimization of the PROPEL Mini stents, with the goal of solving the clinical challenge of postoperative displacement from a mechanical perspective.

## 2. Materials and Methods

### 2.1. Model Construction

**Figure 1:**
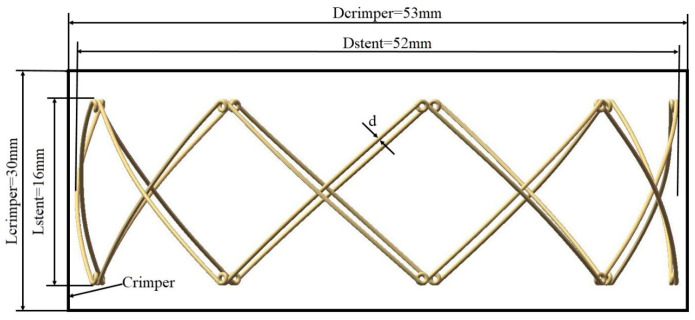
Geometric representations of the PROPEL stent individually and its synchronized assembly within the constraint mechanism. The variables D and L denote the nominal diameter and axial length for the stent (subscript ‘stent’) and the crimper (subscript ‘crimper’), respectively, while d represents the cross-sectional diameter of the wire.

**Figure 2:**
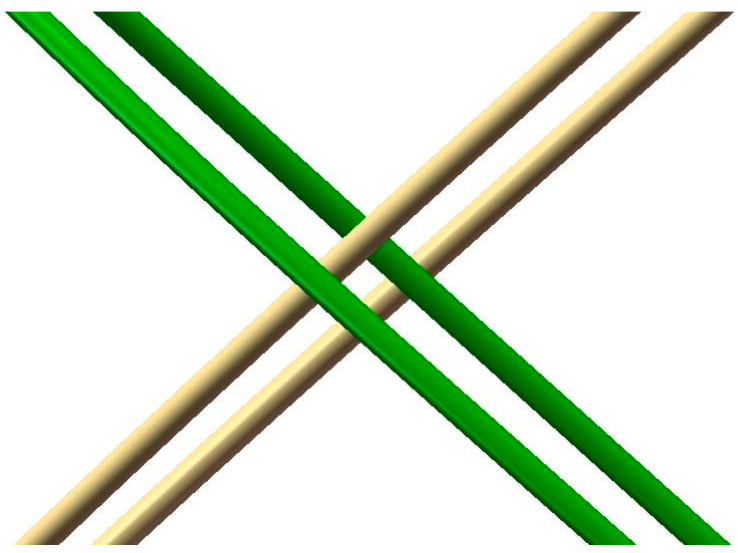
Contact model of the PROPEL stent (To distinguish between the two helical orientations, the right-handed strands are rendered in golden, contrasting with the left-handed strands which are depicted in green).

The geometric model of the PROPEL stent was developed based on the dimensions of the PROPEL mini sinus device (Intersect ENT Inc., Menlo Park, CA, USA). The geometrical model of PROPEL stent was created in SolidWorks 2022 (Dassault Systemes, SolidWorks Corps, Waltham, MA, USA). Then the geometric model in IGES format was imported into ABAQUS. Radial compression and rebound simulations were performed using the Abaqus/Explicit solver (version 6.14; Dassault Systèmes SE, Vélizy-Villacoublay France). The stent geometry was characterized by a wire size of 0.4 mm, an overall diameter of 52 mm, and a total length of 16 mm. For discretization, B31 beam element type was selected; this element type is particularly suited for capturing substantial axial strains and transverse shear effects. Following a mesh convergence study, a 0.08 mm element size was implemented to balance numerical precision with computational efficiency [17]. The geometric architecture of the PROPEL stent and its assembled state with the crimping tool are visualized in Figure 1, while Figure 2 provides a magnified view of the contact mechanics between the overlapping beams. As shown in Figure 2, the golden and green strands are characterized by a consistent braid angle. For the numerical simulation, the crimper was idealized as a rigid cylindrical surface, assigned an internal diameter of 53 mm to accommodate the stent, and an axial span of 30 mm to ensure full coverage. In terms of discretization, we employed M3D4R (reduced-integration membrane) elements. This specific element type was chosen for the crimper because it offers an excellent balance between contact accuracy and computational efficiency in explicit analysis [17]. A refined mesh size of 0.1 mm was applied to the crimper to prevent numerical penetration during the contact interactions with the stent struts.

### 2.2. Numerical Simulation Strategy and Boundary Constraints

Due to the inherently interwoven architecture of the device, the mechanical response during radial crimping and rebound is characterized by complex local interactions at the filament junctions. Specifically, the individual wires are not rigidly fixed but are instead prone to relative rotation and translational sliding at their points of intersection. To accurately capture this behavior in the numerical model, appropriate contact properties and degrees of freedom must be accounted for to reflect the structural flexibility of the braided wires. To facilitate the radial compression and rebound analysis, a local cylindrical coordinate system was established as the primary reference frame. To ensure the numerical stability of the simulation and prevent non-physical rigid body translations or rotations, specific kinematic constraints were imposed on the node located at the longitudinal midpoint of the stent wire. Specifically, the degrees of freedom corresponding to the circumferential (U_2_) and axial (U_3_) directions were restricted to zero (U_2_ = U_3_ = 0). In contrast, the radial displacement (U_1_) remained unconstrained, allowing the stent to deform freely under the prescribed motion of the crimping tool. By pinning the central node, the model is effectively anchored in space without over-constraining its radial expansion or contraction capabilities, thereby allowing for a purely symmetric deformation during the loading sequence.

The movement of the stent was driven by imposing prescribed radial displacement boundary conditions directly onto the crimper’s surface. This motion was governed by a local cylindrical coordinate system, ensuring that the movement was uniformly distributed toward the central longitudinal axis, thus accurately replicating the mechanical crimping and rebound procedure. The entire process of the calculation is divided into two steps: the first is radial compression, and the second is radial rebound. The displacement was applied using a smooth-step amplitude curve in Abaqus/Explicit. In the radial compression step, a smooth step amplitude is applied, varying from 0 at the beginning of the step to 1 at the end. In the radial rebound step, the amplitude of the smoothed amplitude curve is 1 at the beginning, and the amplitude of the smoothed amplitude curve is 0 at the end of the rebound step. This approach was chosen to minimize the generation of unwanted high-frequency oscillations throughout the crimping and rebound phase. During the crimping process, the inner diameter of the crimper was compressed from 52 mm to 5.5 mm. During the release process, the inner diameter of the crimper expands from 5.5 mm to 52 mm (If significant plastic deformation of the stent occurs during compression, the stent will not recover to its original diameter during the release process). The range of 52 mm to 5.5 mm primarily corresponds to the extreme geometric compression experienced by the PROPEL Mini stent in the delivery system. This compression ensures its smooth passage through narrow catheter tips and intraoperative positioning (by increasing its nominal diameter through the recovery of elastic deformation). In actual clinical applications, once the stent is deployed to the target sinus cavity/anatomical location, its radial expansion is mainly constrained by the anatomical dimensions of the lesion site and the elasticity of the surrounding soft tissue, and it will not undergo such significant compression and rebound again.

To accurately simulate the mechanical response during the radial movement phase, the computational model incorporates two categories of contact interactions within the stent–crimper assembly. The first category involves ‘inter-wire contact,’ which governs the intricate frictional interactions and sliding behavior between the overlapping braided filaments as the structure densifies. The second category is the ‘stent-to-tool’ interface contact, established between the advancing internal luminal surface of the cylindrical crimper and the external boundary of the PROPEL stent. Both contact pairs are essential for ensuring that the load is uniformly transmitted from the tool to the device or from the device to the tool without numerical penetration. Regarding contact management, the ‘General Contact’ algorithm within Abaqus/Explicit was implemented to govern the interactions across the entire assembly. This choice was dictated by the solver’s ability to automatically track and define complex contact pairs, which significantly enhances the robustness of the simulation compared to manual contact pair definitions [17]. Furthermore, as the radial movement is inherently a slow process, it was modeled as a quasi-static event. Achieving this quasi-static event required a ‘smooth step’ amplitude function to apply the loading gradually, avoiding sudden velocity jumps. Initially, shorter step times (e.g., 0.1 s or 0.2 s) were tested, but they resulted in excessive kinetic energy spikes. Increasing the duration to 0.768 s provided a sufficiently smooth response while maintaining a reasonable computational wall-clock time. For the interfacial physics, the tangential behavior was modeled using Coulomb friction, with a coefficient of 0.1 applied to inter-wire interactions based on established literature [17,18,19,20]. In contrast, the contact between the crimper and the stent was defined as frictionless (μ = 0) to simplify the radial force transmission analysis. Double precision was used in all simulations, and no mass scaling was applied. General contact with penalty-based enforcement was defined between the stent wires and the rigid crimper, including self-contact among wires.

The mechanical properties of the constituent materials for the PROPEL stent, which include the biodegradable polymers poly (lactic-co-glycolic acid) (PLGA) and polycaprolactone (PCL), as well as a biocompatible Mg alloy (Mg-2.2 Nd-0.1 Zn-0.4 Zr (wt.%, denoted as JDBM-2)), were sourced from established literature [8,21,22]. These detailed material parameters, essential for the constitutive modeling in our simulation, are presented in Table 1. Although only the Young’s moduli are summarized in Table 1 for comparison, elastoplastic material models with yield stress and post-yield laws were implemented for all materials based on literature-reported stress–strain curves [8,21,22]. The corresponding material curves are provided in Supplementary Figure S1. During extreme compression to the minimum diameter, plastic deformation was observed in the PLGA and Mg stents, whereas the PCL stent remained fully elastic.

## 3. Results

### 3.1. Radial Pressure–Diameter Characteristics

**Figure 3:**
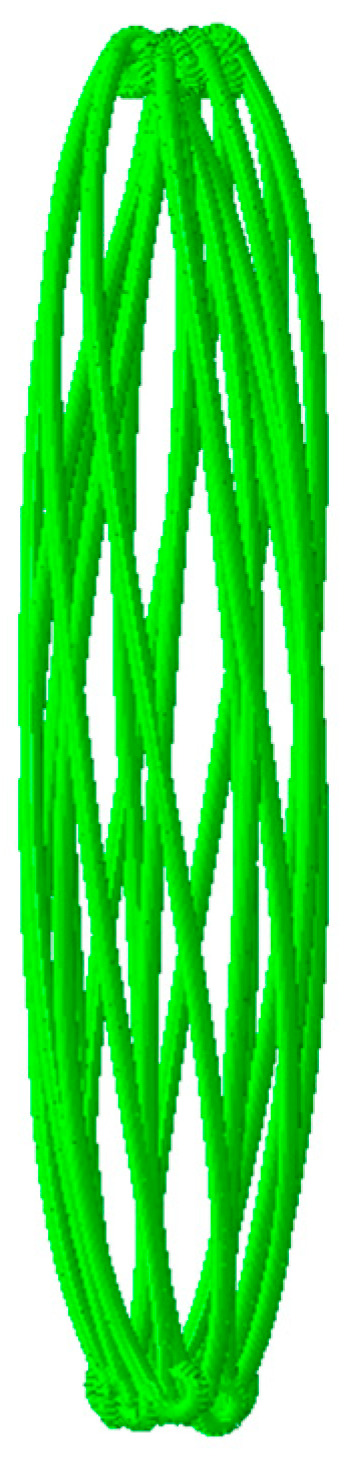
PROPEL stent after crimping.

**Figure 4:**
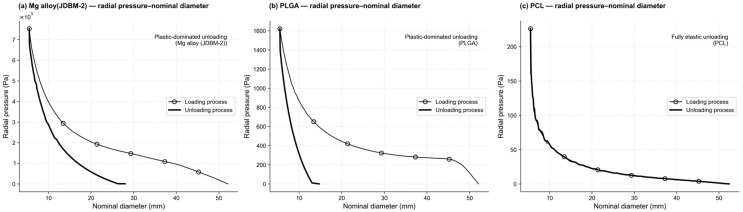
Relationship between the radial pressure and nominal diameter of the stent in the process of loading and unloading.

Three distinct material configurations were evaluated using finite element modeling. The structural response of the PROPEL stent subjected to uniform radial loading is depicted in Figure 3. Consistent with findings for open-ended braided designs [13], the observed radial deformation exhibits longitudinal heterogeneity. This indicates that the stiffness of PROPEL stent is also non-uniform along the axial direction. In clinical practice, the magnitude of the radial pressure exerted on the surrounding tissues after stent deployment is very important, as it directly relates to the stability and reliability of the implanted stent. The relationship between the radial pressure and nominal diameter of the stent in the released process is presented in Figure 4.

As shown in Figure 4a,b, during the recoil process, the stent made of Mg alloy recoiled from a diameter of 5.5 mm to 28 mm; the stent made of PLGA recoiled from 5.5 mm to 15 mm approximately. This indicates that the stents made from these two materials underwent plastic deformation during compression, which prevented them from fully returning to their initial diameter during the recoil process. As shown in Figure 4c, during the recoil process, the diameter of the PCL stent rebounded from 5.5 mm to 52 mm. This indicates that the stent made of PCL fully recovers to its initial diameter during the recoil process. In fact, the PCL stent undergoes elastic deformation throughout the entire compression and rebound process (during the two processes, its equivalent plastic strain value is zero). For the three materials, as the nominal diameter increases, the radial pressure decreases. However, the stents made of Mg alloy and PLGA exhibit significantly higher released radial pressures than the PCL stent during unloading, but this occurs over a comparatively low diameter range due to plastic deformation, as their post-release recovery is limited to approximately 28 mm and 14 mm, respectively, whereas the fully elastic PCL stent can recover to its original nominal diameter (52 mm) with a much lower radial pressure. This issue will be discussed in detail in the Section 4.

**Figure 5:**
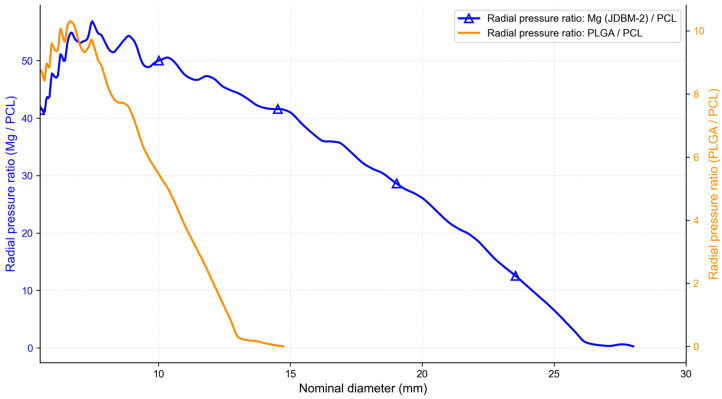
Radial pressure ratio–diameter characteristics of the stent in the released process.

The PROPEL Mini stent made of PLGA has received FDA clearance for clinical use. To compare the difference between the radial pressure during rebound of the stent made of Mg alloy, PLGA and PCL, the relationships between the radial pressure ratio and nominal diameter are shown in Figure 5. The typical diameter range of the ethmoid sinus cavity is 12–15 mm. Within this diameter range, as shown in Figure 5, the ratio of radial pressure between Mg alloy stents and PCL stents is between 40.7 and 46.9, while the radial pressure ratio between the PLGA stent and the PCL stent is between 0.0025 and 2. For example, When the nominal diameter of the stent rebounds to 12 mm, the radial pressure released by the Mg alloy stent is 23.9 times as large as that released by the PLGA stent; the radial pressure released by the PCL stent is 0.5 times as large as that released by the PLGA stent.

### 3.2. Length–Diameter Characteristics of the Stent

**Figure 6:**
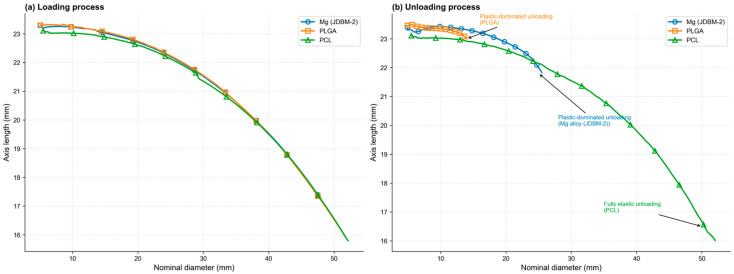
The relationship between nominal diameter and axial length.

As shown in Figure 6a, the compression deformation curves between the axial length and nominal diameter of the PROPEL stent with three different materials are almost identical. Reducing the PROPEL stent’s nominal diameter from 52 mm to 5.6 mm results in an axial elongation from 16 mm to approximately 23 mm. As shown in Figure 6b, when the stent made of Mg alloy rebounds from a nominal diameter of 5.5 mm to 28 mm during the unloading process, its axial length decreases from approximately 23 mm to approximately 22 mm. When the PLGA stent rebounds from a nominal diameter of 5.5 mm to 14 mm during the unloading process, its axial length decreases slightly from approximately 23.5 mm to approximately 23 mm. Because the PCL stent does not undergo plastic deformation during loading and unloading, when its nominal diameter rebounds from 5.5 mm to 52 mm during unloading, its axial length decreases from 23 mm to 16 mm, recovering its initial geometric shape.

### 3.3. The Ratio Between Internal Energy and Kinetic Energy of the Stent

**Figure 7:**
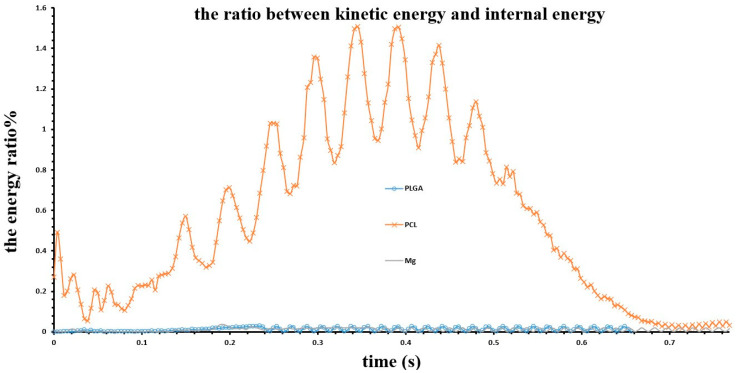
The ratio between kinetic energy and internal energy of the stent with different materials in the released process.

To ensure the accuracy and reliability of the calculation results, the simulation calculation must be quasi-static. The validity of the quasi-static solution was confirmed by maintaining the stent’s kinetic energy within 5% of its internal energy throughout the radial loading. As shown in Supplementary Figure S2, at the initial crimping stage of the contact between the crimper and the stent (from 0.096 s to 0.1152 s), the ratio between kinetic energy and internal energy of the stent is greater than 0.05; however, after 0.1152 s, the crimper crimped the stent, and the kinetic-to-internal energy ratio of the stent with different materials is less than 0.05. As shown in Figure 7, during the unloading process, the ratio of kinetic energy to internal energy of the stents made of the 3 materials remained consistently below 1.6%. This confirms the quasistatic nature of the simulation.

## 4. Discussion

Lu et al. [8] highlighted that the sinus cavity’s irregular geometry increases the potential for stent dislodgement. Such displacement toward the cranial base represents a severe clinical hazard [23]. Consequently, quantifying the interfacial friction between the stent and the sinus wall is essential for assessing migration resistance. If the friction force is high, the stent has a strong ability to resist migration.

In accordance with the fundamental principles of the Coulomb friction law, the magnitude of the frictional resistance generated at the interface is directly proportional to the normal force acting between the two interacting surfaces. Under the same friction coefficient, the greater the normal force, the greater the friction force on the stent. After the implantation of the stent is completed, the normal force exerted by the stent on the surrounding tissue is calculated by integrating the radial pressure on the cylindrical contact surface. Under the assumption of an idealized cylindrical constraint, this normal force can be expressed as the product of the radial pressure and the lateral surface area of the stent, i.e., *F_n_* = *p_r_*·*πDL*, where *p_r_* denotes the radial pressure, *D* is the nominal stent diameter, and *L* is the axial length. When the contact area is constant, the greater the radial pressure, the greater the normal force, and hence the greater the frictional force.

At the same time, it should be noted that radial pressure in the present study is used as a comparative mechanical indicator rather than a direct predictor of in vivo anchoring, given the simplified contact assumptions and the complex anatomy of the sinus cavity. The purpose of the research is to compare the relative mechanical response of stents fabricated from different materials under identical loading and boundary conditions, rather than to fully reproduce the complex anchoring behavior in real sinus anatomy. Anchoring of sinus stents in vivo is influenced by multiple factors beyond radial pressure alone, including local anatomical irregularity, mucosal compliance, surface roughness, tissue remodeling, and non-uniform contact between the stent and sinus walls. These effects are not explicitly captured in the present model. Nevertheless, previous studies on self-expanding stents have consistently used radial force or radial pressure as a first-order mechanical indicator of anchoring tendency, particularly for comparative or parametric studies under controlled assumptions. Within this context, a higher radial pressure can be interpreted as an increased mechanical potential for anchoring, rather than a definitive clinical outcome.

As shown in Figure 4 and Figure 5, during stent’s unloading, the radial pressure released by the stent made of Mg alloy is greater than that released by the stent made of PLGA (within the nominal stent diameter range of 5.5 mm to 14 mm). The PCL stent releases much lower radial pressure than PLGA; however, unlike PLGA, which provides negligible support beyond 14–15 mm due to plastic deformation, PCL remains elastically active and continues to exert a small residual pressure at larger diameters. While experimental radial pressure data for the PROPEL Mini stent are not available in the literature, previous experimental and numerical studies on braided self-expanding stents report radial resistive forces of several newtons at small diameters [24]. Normalizing these forces by the stent–wall contact area yields pressure levels in the same order of magnitude as those obtained in the present simulations. The observed material-dependent differences in radial pressure are consistent with reported trends for polymeric and metallic braided stents. Our results are in agreement with the findings of Alok Srivastava et al. [25], who reported that Mg alloy stents can generate higher radial force than polymer-based stents. In our unloading simulations, the Mg stent indeed releases substantially higher radial pressure than PLGA within the recovered diameter range; however, its elastic recovery is limited to approximately 28 mm due to plastic deformation, whereas the fully elastic PCL stent can maintain a small but non-zero radial pressure at larger diameters beyond this range.

Sinus stent stability relies on a robust interference fit within the surgical cavity. Our simulations show that while Mg alloy exhibits a permanent set, its nominal recovery to 28 mm is significantly higher than the 15 mm limit of PLGA. Conversely, PCL achieves full recovery (52 mm) but offers clinically inadequate radial pressure (the maximum radial pressure is less than 165 Pa).

Clinical morphometric data confirm that the 28 mm nominal recovery of the magnesium (Mg) alloy stent is highly compatible with post-FESS anatomical targets. Although the vertical elevation of the posterior ethmoid roof is approximately 44.64 mm from the nasal floor, this value represents a spatial landmark rather than the actual internal cavity height, which typically ranges from 10 to 15 mm [26]. Furthermore, critical stenotic areas like the frontal ostium exhibit mean diameters of only 7.22 ± 2.78 mm and 8.92 ± 2.95 mm [27]. While we acknowledge that cavity dimensions may occasionally exceed 28 mm in rare anatomical variants or following extensive skull base surgery, stenting is primarily indicated for maintaining patency in narrowed or stenotic passages. In these scenarios, the high radial stiffness of the Mg alloy ensures a robust interference fit against the sinus walls, providing a more reliable solution to the accidental expulsion and displacement issues inherent in lower-strength polymer designs [9,21].

Our simulations showed that the PLGA stent recovered to only 15 mm, significantly lower than the 40 mm recovery reported by Medtronic. This discrepancy is likely due to the limitation of using a time-independent elastoplastic model. PLGA is a semi-crystalline polymer exhibiting pronounced viscoelasticity and shape-memory effects, which are highly sensitive to body temperature (37 °C) and hydration [11,14]. As investigated by Bobel et al. [14] and Cheng et al. [28], polymer stents undergo significant stress relaxation and gradual geometric recovery over time post-implantation. Consequently, the 15 mm result captures only the instantaneous elastic–plastic rebound, representing the stent’s initial state. The manufacturer’s reported expansion likely reflects the long-term, time-dependent recovery driven by polymer chain mobility, a process that requires the incorporation of complex viscoelastic subroutines in future computational models.

A clinical case report by Kevin J. Choi et al. clearly documented a patient who experienced immediate restenosis after the stent’s implantation [29]. Although it was only one case (out of five patients included, accounting for 20%), it undoubtedly indicated insufficient support from the PROPEL Mini stent. To avoid this situation, Mg alloy stents may be a good alternative. But this does not mean that PLGA stents should be completely replaced by Mg alloy stents because stent migration or immediate stenosis after interventional surgery does not necessarily occur. However, determining which stent to use before surgery requires the accumulation of a large amount of clinical data. Furthermore, Mg alloy stents provide strong support and have a long degradation time. This leads to the concern of restenosis during the longer degradation period.

Although this study revealed the initial mechanical response of stents made of different materials, it still has the following limitations. First, both PLGA and PCL were modeled as elastic–plastic materials to evaluate their short-term mechanical response during quasi-static compression and immediate post-deployment expansion. Therefore, the reported radial pressures represent instantaneous mechanical support rather than long-term sustained forces after stress relaxation [30]. However, for polymer braided structures like PROPEL Mini, the time-dependent mechanical response cannot be overlooked. As investigated by Cheng et al. [28], PLLA braided stents exhibit significant stress relaxation shortly after deployment, which can lead to a change in the effective radial supporting force. Incorporating such viscoelastic properties in future models would provide a more accurate estimation of long-term stent stability in the sinus cavity. Second, the study only simulated the static mechanical state at the initial stage and did not consider the dynamic degradation process of materials under physiological humid and hot conditions. Although Mg alloys have shown the potential to maintain long-term radial strength better than polymers due to their lower degradation rate than PLGA, this model cannot quantitatively predict the difference in strength evolution between the two materials during the degradation cycle [31]. Finally, the study used an idealized cylindrical constraint, which failed to fully reproduce the non-uniform stress distribution and local buckling risk caused by the irregular geometric anatomy of the real sinus cavity [32]. Previous research by Djukic et al. [33] emphasized that simulations using patient-specific anatomical geometries often reveal localized stress concentrations and displacement risks that are absent in simplified models. Therefore, future work involving patient-specific sinus CAD models is necessary to validate the performance of these material models under irregular anatomical constraints. While this study confirms the advantage of Mg alloys in initial radial strength, combined with their slower degradation rate, Mg alloy stents hold promise for providing more durable mechanical support during the critical postoperative window. Future research should incorporate damage mechanics models to further validate their mechanical stability advantages throughout the entire degradation cycle.

## 5. Conclusions

This study addresses the issues of displacement and insufficient stability of the PROPEL Mini stent in clinical practice by systematically analyzing the mechanical response of the monofilament braided structure under different material constraints. While axial elongation is primarily governed by geometric topology during loading, material-specific plasticity leads to a permanent geometric set in Mg alloy and PLGA models upon unloading. Nevertheless, the Mg alloy stent provides a radial supporting capacity that exceeds that of traditional polymers, ensuring robust scaffolding within the typical anatomical dimensions of the sinus cavity. This discovery not only fills a theoretical gap in the mechanical mapping relationship of closed-cell braided stents, but also demonstrates the feasibility of the introduction’s idea of solving clinical problems through material innovation. The study concludes that, without altering the existing non-invasive structure, introducing high-strength biodegradable Mg alloys is the most effective scientific approach to enhance the radial anchoring force of the stent and improve postoperative positional stability, providing theoretical guidance for the future development of high-performance sinus stents.

## Figures and Tables

**Table 1:** Mechanical characteristics of PLGA, PCL, and Mg alloy.

Constituent	Elastic Modulus (MPa)	Mass Density (kg/m^3^)	Yield Strength (MPa)	Poisson’s Ratio
PLGA	26,552	1300	55	0.36
PCL	289	1140	12	0.30
Mg alloy	44,843	1740	312	0.30
Crimper	—	6450	—	—

## Data Availability

The original contributions presented in the study are included in the article; further inquiries can be directed to the corresponding author.

## Supplementary Materials

The following supporting information can be downloaded at: https://www.mdpi.com/article/10.3390/jfb17020083/s1, Figure S1: Elastoplastic material models used in the finite element simulations; Figure S2: the ratio between kinetic energy and internal energy of the stent with different materials in the crimping process.
