# Supplementary figures and images for: Comparative Finite Element Evaluation of Polymeric and Metallic Bioresorbable Sinus Stents Under Quasi-Static Radial Compression
Source: J Funct Biomater. 2026 Feb 8;17(2):83. doi: 10.3390/jfb17020083 (PMC12941876; doi:10.3390/jfb17020083)

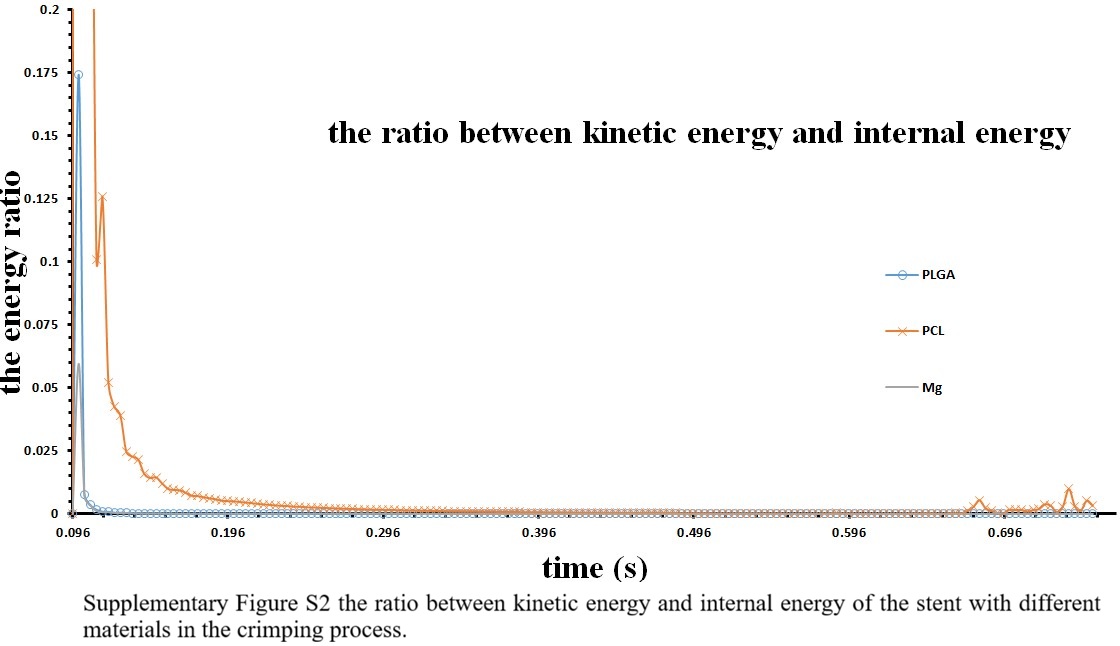

Supplement: Supplementary file 1 [file jfb-17-00083-s001.zip › Supplementary Figure S2 the ratio between kinetic energy and internal energy of the stent with different materials in the crimping process..JPG]

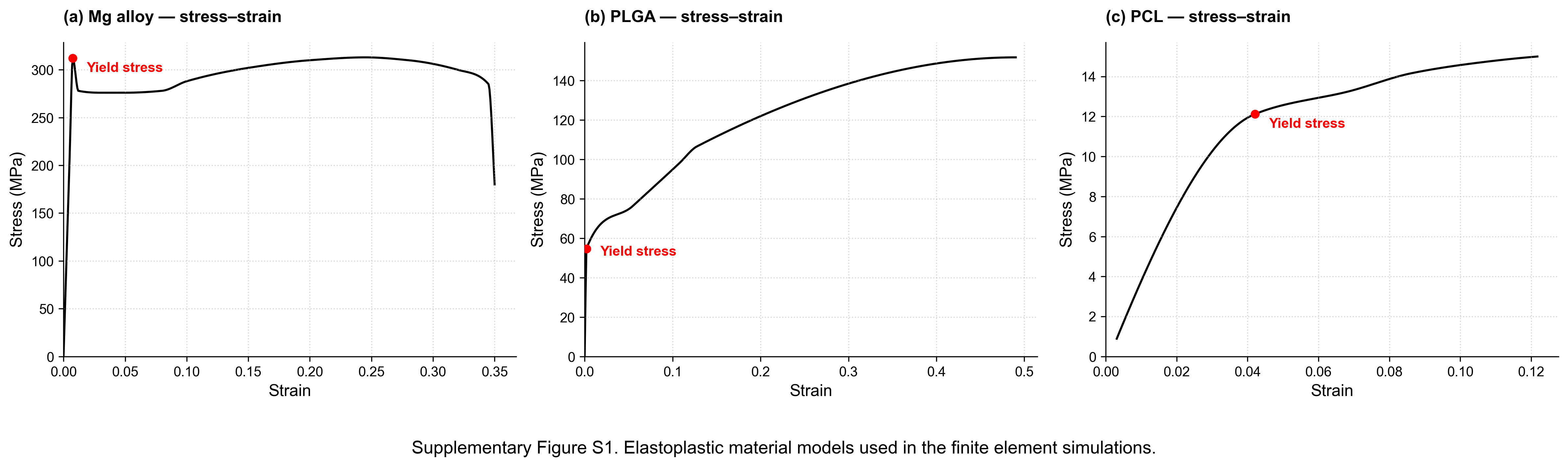

Supplement: Supplementary file 1 [file jfb-17-00083-s001.zip › Supplementary_Figure_S1_Pchip.png]
